# 

**Published:** 1908-04

**Authors:** 


[P]UBLISHED MONTHLY. ATLANTA SUBSCRIPTION $1.[illegible]

### JOURNAL-RECORD OF MEDICINE

[Succ]essor to { Atlanta Medical and Surgical Journal, Established 1855. and Southern Medical Record, Established 1870. } 52d Year

[V]ol. X. Atlanta, Ga., April, 1908. No. 1

